# Resistin as a risk factor for all-cause (and cardiovascular) death in the general population

**DOI:** 10.1038/s41598-022-24039-2

**Published:** 2022-11-15

**Authors:** María del Cristo Rodríguez Pérez, Delia Almeida González, Itahisa Marcelino Rodríguez, Santiago Domínguez Coello, Francisco J. Cuevas Fernández, Buenaventura Brito Díaz, Antonio Cabrera de León

**Affiliations:** 1 Unidad de Investigación de Atención Primaria y del Hospital Universitario Nuestra Señora de Candelaria, Santa Cruz de Tenerife, Spain; 2 Unidad de Investigación, Hospital de La Candelaria, Carretera de El Rosario 145, 38010 Santa Cruz de Tenerife, Canary Islands Spain; 3 Área de Medicina Preventiva y Salud Pública, Universidad de La Laguna, La Laguna, Spain

**Keywords:** Immunology, Biomarkers, Medical research, Risk factors

## Abstract

Serum resistin is a pro-inflammatory cytokine that has been described as a risk factor associated with mortality in several clinical sets including type 2 diabetes. Mortality studies in the general population are needed to find out the risk of death associated to this cytokine. In a follow-up study of a cohort of adult population (n = 6636) in Spain over a period of fifteen years (447 deaths/102,255 person-years), serum resistin measurements and death records were obtained. The risks of all-cause deaths, and deaths from cardiovascular and oncological diseases were estimated. Hazard ratios (HR) and its confidence intervals (CI) were calculated using multivariable Cox models, adjusting the effect of 11 traditional risk factors. The risk of all-cause mortality among participants exposed to the highest quintile of resistin was always higher than among those in the lowest quintile (HR varied between 1.55 when smoking was the adjusted factor [95% CI 1.17–2.05], and 1.68 when the adjusted factor was physical activity [95% CI 1.27–2.21]). The maximally adjusted model, accounting for the effect of all traditional factors, corroborated this higher risk of all-cause mortality among people in the highest resistin quintile (HR = 1.52; 95% CI 1.13–2.05). The effect of resistin was even higher for cardiovascular deaths (HR = 2.14; 95% CI 1.13–4.06), being exceeded only by suffering diabetes (HR = 3.04; 95% CI 1.98–4.69) or previous acute coronary syndrome (HR = 3.67; 95% CI 2.18–6.18). This findings corroborate the role of resistin as a risk factor for all-cause (and cardiovascular) death in the general population.

## Introduction

Resistin was discovered at the beginning of the twenty-first century as a molecule produced by the adipocytes of mice and a role as a mediating hormone between diabetes and obesity through insulin resistance was proposed for it^1^. It was found that premature coronary artery disease patients exhibited increased serum levels of resistin, and a possible mechanism by which resistin might contribute to atherogenesis was suggested^2^. In the human species, resistin turned out to be a pro-inflammatory cytokine secreted mainly by macrophages^3^ and which, in the general population, is directly associated with the appearance of coronary heart disease^4^ and inversely associated with adherence to the Mediterranean diet^5^ and, with physical activity and HDL-cholesterol^6^. Generally speaking, few individuals have been analyzed in resistin studies, with meta-analyzes being used to corroborate its association with coronary heart disease^7^. Although controversy persists regarding its relationship with diabetes or insulin resistance^8^. One of the few follow-up studies recruited 599 aged people and concluded that resistin is a risk factor for mortality in the aged population^9^. An inflammatory pathway has recently been described which includes resistin together with other cytokines and which improves the prediction of death in people with type 2 diabetes^10^. Mortality studies in the general population are needed to verify the translational potential of this cytokine as a risk factor.

By following a cohort of the general population in Spain over a period of fifteen years, the objective of this study is to assess the risk of death associated to serum resistin concentrations, adjusting the effect of traditional factors that have been related with it and determinants of mortality such as diabetes, hypertension, social class or smoking.

## Methods

### Study population and design

The participants of this study were from the cohort “CDC de Canarias” (CDC is the acronym for Cardiovascular, Diabetes, Cancer), which enrolled randomly selected adult individuals from the general population of the Canary Islands, in Spain, between 2000 and 2005. The cohort (n = 6729 subjects) constituted a representative sample of the population aged eighteen to seventy-five years at the time of recruitment.

The design and methods used in the “CDC de Canarias” cohort have been described previously^11^ and their previous results with resistin have been published^4–6^. The study was approved by the Ethics and Medicines Committee of the Hospital Universitario Nuestra Señora de Candelaria (HUNSC). The participation rate was 70%. All research was performed in accordance with the Declaration of Helsinki and relevant guidelines or regulations, and the informed consent was obtained from all participants or their legal guardians.

### Exposure factors

Each person was interviewed about their personal and family health and lifestyle history (diet, physical activity, smoking, education, family income and living conditions). In addition, a physical examination was performed, which analyzed blood pressure (measured using a previously calibrated sphygmomanometer, with the subject seated and having been at rest for 5 min. Two measurements were taken and their average was used for the study) and body mass index (BMI, categorized as normal [< 25 kg/m^2^], overweight [25–29 kg/m^2^] or obesity [≥ 30 kg/m^2^]), and a venous blood sample was obtained after ten hours of fasting.

The samples were transferred daily to the HUNSC laboratory, after in situ centrifugation, for biochemical measurements within twenty-four hours; HDL-cholesterol (HDL) was measured with a Hitachi 917 auto analyzer (spectrophotometric methods) and was categorized as low when it presented values < 40 mg/dL in men or < 50 mg/dL in women. Resistin was measured in 6636 participants: Serum aliquots were frozen at − 80 °C until their use for the measurement of resistin by enzyme-immunoassay (ng/mL, Bio-Vendor^®^, Heidelberg, Germany; inter-assay variation coefficient = 7.72%; and intra- trial = 3.22%)^5^; quintiles (Q) were used to categorize it for the mortality analysis.

*Dietary habits* were obtained with a questionnaire of frequency and quantity of intake previously validated in the study population^12^; the scale proposed by Trichopoulo^13^ was used and adherence quintiles were calculated to analyze adherence to the Mediterranean diet. The data on *physical activity* were obtained with the Spanish version of the Minnesota Questionnaire on Physical Activity in Leisure Time^14^, and each activity described by the participants was assigned a value in metabolic equivalents (MET), according to the Compendium of Physical Activities by Ainsworth et al.^15^ This activity was measured using the quotient between moderate or intense physical activity (four or more MET) and total daily energy expenditure, as described by Bernstein et al.^16^; the quintiles of this quotient were calculated taking quintile 1 (Q1) as sedentary.

The ICE (Income, Crowding, Education) model which had been validated in this population^17^ was used to measure the *social class* of the participants. The ICE produces a numerical scale with a range of values between 4 and 21, where the higher the social class, the higher its value is; their quintiles were calculated for the mortality analysis.

The participant's declaration of suffering diabetes and receiving antidiabetic treatment was accepted as *diabetes* for the purposes of the study; otherwise, a fasting blood glucose level of ≥ 126 mg/dL was required, corroborated in a second analysis by his family doctor. *Hypertension* was recorded when the participants declared that they had the disease and were receiving antihypertensive treatment, or if the participants did not know they had hypertension but the mean of two blood pressure readings was ≥ 140 mmHg for systolic or ≥ 90 mmHg for diastolic blood pressure. *Dyslipidemia* was recorded when there was a previous diagnosis and the participants declared they were receiving treatment, or when they had fasting serum levels ≥ 240 mg/dL of total cholesterol. Participants who declared that they smoked at least one cigarette per day were considered *smokers*. *Alcohol* consumption was classified as abstemious if the declaration was less than 1.5 g/day, moderate if the intake was between 1.5 and 30 g/day and high if the consumption was greater than 30 g/day.

*Acute coronary syndrome* cases were recorded when the participant declared that they had experienced an acute myocardial infarction or angina pectoris and this information was verified in the digital medical records of the patient in their public primary care center or hospital, with the permission of the participant.

### Mortality registry

The identification of the participants who died during the follow-up of the cohort, as well as the cause and date of death, were obtained from the National Institute of Statistics, which in Spain collects the information obtained from all medical death certificates and uses the diagnostic codes of the 10th Revision of the International Classification of Diseases. In addition to all-cause deaths, the following three large groups of causes were analyzed: oncological (C00–C97; D00–D09 and D37–D48), cardiovascular (I00–I09, including diabetes [E10–E14]) and a third group for all other causes.

### Statistical analysis

The numerical variables were summarized with their mean (± SD) and the nominal variables with the absolute and relative frequency of their component categories. The baseline comparisons between the resistin quintiles were made with a chi-square test for trends.

The association of death with exposure to the factors studied was analyzed with Cox proportional hazards regression models, both for all-cause mortality and for the three large groups of causes of death. The follow-up time of each participant was counted from the initial recruitment interview until December 31, 2019 or the date of death. Resistin was first included as a continuous variable, then transformed by taking its squared root to improve its approximation to the normal distribution and, finally, categorized into quintiles. The results are summarized with the hazard ratio between the categories of each factor and their 95% confidence interval (HR, 95% CI).

One model was adjusted by age and sex for resistin and another for each of the eleven exposure factors studied (hypertension, diabetes, acute coronary syndrome, BMI, smoking, alcohol, Mediterranean diet, social class, physical activity, dyslipidemia, and HDL cholesterol). The effect of resistin was then adjusted in eleven models, each of which included, in addition to age and sex, one of the exposure factors. Finally, this effect was analyzed using a maximally adjusted model that included all the factors; the survival function of resistin quintiles obtained in this model for the all-cause deaths is offered as a figure, and the resistin HR values (95% CI) at 5, 10 and 15 years of follow-up were used to create a sequence graph that is also shown as a figure. One more model was adjusted including, in addition to the factors, the treatment with statins, antihypertensives, and antidiabetics.

The assumption of proportional hazards was tested by regression of the logarithm of the negative logarithm of the estimated survival function against the logarithm of time, with visual evaluation of the linearity in the graph between both variables, and checking that the product-terms between the factors were not significant when they were included in the models together with the follow-up time (log-transformed).

The variable with the highest number of missing values was social class (345 participants [5.2% of the cohort]), with missing values being less than 1% in the other factors studied. In a sensitivity analysis, social class was substituted for the individual's educational level, which is the main component of the ICE model (only fifteen participants did not have this data [0.2%]). There were also ninety-three participants (1.4% of the cohort) with no resistin determination at recruitment. In a final sensitivity analysis we used a multiple-imputation procedure to impute values for missing data on social class and resistin before making categories. The studied variables were included as covariates in the multiple imputation model. To impute missing values, the Markov Chain Monte Carlo based method was used; to compute the HR and their 95% CI from the imputed dataset, the estimated beta coefficients were averaged and exponentiated.

All hypothesis contrast tests used were two-tailed and p values less than 0.05 were considered statistically significant. The analyzes were performed with the computerized statistical data processing package SPSS^®^, version 24.0 in Spanish.

## Results

### Characteristics of the participants

The 6636 people who were measured for resistin were followed for 15.4 ± 2.4 years, during which time 447 of them died; of these, 193 were deaths due to cancer, 108 due to cardiovascular causes, and 146 due to other causes. Table 1 summarizes the characteristics of the participants when they were enrolled in the cohort, according to sex and resistin quintiles, and shows the distribution of the 102,255 person-years of follow-up in these groups.

**Table 1:** Number and characteristics of the participants at the time of enrollment, according to sex and quintiles of resistin.

	Sex		Resistin					P for trend
	Women	Men	Q1	Q2	Q3	Q4	Q5	
n	3752	2884	1344	1320	1318	1334	1320	NA
Persons-year	58 596	43 659	20 918	19 990	20 272	20 653	20 422	NA
Resistin (ng/mL)	**6.1 ± 2.4**	**5.6 ± 2.2**	**3.6 ± 0.6**	**4.6 ± 0.2**	**5.4 ± 0.2**	**6.3 ± 0.4**	**9.4 ± 2.6**	**NA**
Age (years)	42.9 ± 12.8	42.9 ± 12.8	46.5 ± 12.8	43.7 ± 12.8	41.7 ± 12.6	41.7 ± 12.8	41.0 ± 12.2	< 0.001^b^
Male sex %	NA	NA	52.2	45.4	44.1	38.8	36.7	< 0.001
BMI (kg/m^2^)	27.3 ± 5.5	27.5 ± 4.2	27.9 ± 4.7	27.3 ± 4.9	27.2 ± 4.9	27.4 ± 5.2	27.2 ± 5.4	< 0.001^b^
Total physical activity (MET/day)^a^	27.0(25.6–30.0)	28.5(26.0–37.8)	27.9(25.8–31.8)	28.0(25.9–32.4)	27.7(25.7–31.9)	27.4(25.6–31.3)	27.3(25.6–30.7)	< 0.001^b^
HDL cholesterol (mg/dL)	54.7 ± 13.1	46.4 ± 12.4	52.0 ± 13.9	50.9 ± 13.5	50.9 ± 12.8	51.4 ± 13.3	50.4 ± 13.8	0.033
Mediterranean diet scale	4.5 ± 1.6	5.0 ± 1.6	4.9 ± 1.5	4.7 ± 1.6	4.8 ± 1.6	4.6 ± 1.6	4.6 ± 1.6	< 0.001^b^
Alcohol ≤ 1.5 g/day %	71.3	29.7	47.6	52.9	54.2	56.0	55.7	< 0.001
1.5 g/day ≤ Alcohol ≤ 30 g/day %	27.9	51.7	40.9	38.1	37.9	37.3	37.0	
Alcohol > 30 g/d %	0.8	18.6	11.5	9.1	7.9	6.8	7.3	
Social class	13.4 ± 3.4	13.3 ± 3.5	13.1 ± 3.4	13.2 ± 3.4	13.4 ± 3.4	13.5 ± 3.5	13.5 ± 3.4	< 0.001^b^
Smoking %	21.6	31.3	20.0	23.1	24.8	27.6	33.5	< 0.001
Dyslipidemia %	30.8	32.3	37.3	31.0	28.3	28.1	28.5	< 0.001
Hypertension %	31.3	39.5	41.3	37.9	33.5	32.9	28.6	< 0.001
Diabetes %	9.5	11.8	14.2	9.6	11.1	8.4	9.1	< 0.001
Acute coronary syndrome %	1.8	3.5	2.9	2.8	2.2	2.3	2.4	> 0.05

^a^Median and interquartile range.

^b^Trends are for quintiles of these variables across resistin quintiles.

Resistin values are in bold.

At the time of recruitment (Table 1), the group exposed to resistin Q5 had the highest proportion of women (63.3%) and the highest prevalence of smoking (33.5%); but it was the one with the youngest age (41 years), and lowest prevalence of: hypertension (28.6%), alcohol consumption (55.7% abstainers), physical activity (27.3 MET/day), dyslipidemia (28.5%), and serum HDL cholesterol concentration (50.4 mg/dL).

### Resistin and mortality

In Table 2 the multivariate adjustments that included only resistin plus one of the exposure factors, in addition to age and sex, show that participants exposed to the highest values of resistin had a higher risk of oncological, cardiovascular, and all-cause mortality. Supplementary Table S1 shows a model for each factor and cause of mortality, adjusted for age and sex: resistin was associated with all-cause deaths (HR 1.67 [1.23–2.18] for Q5 vs Q1) and cardiovascular deaths (HR 2.32 [1.24–4.31] for Q4 vs Q1; and HR 2.06 [1.05–4.04] for Q5 vs Q1.

**Table 2:** Association between resistin and mortality. The effect of resistin was adjusted in eleven models for each type of mortality. The models included age and sex, plus one of the exposure factors, and they were summarized with the HR (95% CI) among resistin categories.

Exposure factors	Oncological deaths HR (95% CI)	Cardiovascular deaths HR (95% CI)	Deaths from other causes HR (95% CI)	All-cause deaths HR (95% CI)
Resistin (BMI) Q2 vs Q1	1.04 (0.68–1.61)	1.20 (0.65–2.21)	1.22 (0.76–1.95)	1.13 (0.86–1.51)
Q3 vs Q1	1.05 (0.68–1.65)	1.28 (0.68–2.42)	0.87 (0.51–150)	1.03 (0.76–1.40)
Q4 vs Q1	0.93 (0.59–1.48)	**2.03 (1.18–3.50)**	0.94 (0.56–1.57)	1.15 (0.86–1.54)
Q5 vs Q1	**1.59 (1.05–2.40)**	**1.87 (1.04–3.36)**	1.48 (0.92–2.39)	**1.61 (1.22–2.12)**
Resistin (Phys Act) Q2 vs Q1	1.09 (0.71–1.67)	1.19 (0.65–2.20)	1.23 (0.77–1.97)	1.16 (0.88–1.54)
Q3 vs Q1	1.07 (0.68–1.68)	1.29 (0.68–2.42)	0.94 (0.55–1.61)	1.07 (0.79–1.45)
Q4 vs Q1	0.95 (0.60–1.50)	**2.02 (1.17–3.49)**	0.97 (0.58–1.63)	1.17 (0.88–1.57)
Q5 vs Q1	**1.68 (1.11–2.53)**	**1.85 (1.03–3.33)**	1.55 (0.96–2.50)	**1.68 (1.27–2.21)**
Resistin (HDL) Q2 vs Q1	1.05 (0.68–1.62)	1.18 (0.64–2.18)	1.20 (0.75–1.92)	1.13 (0.85–1.5)
Q3 vs Q1	1.06 (0.67–1.66)	1.28 (0.68–2.42)	0.94 (0.55–1.60)	1.06 (0.78–1.44)
Q4 vs Q1	0.94 (0.60–1.49)	**1.93 (1.12–3.35)**	0.96 (0.57–162)	1.15 (0.86–1.54)
Q5 vs Q1	**1.60 (1.06–2.41)**	**1.82 (1.01–3.29)**	1.47 (0.91–2.37)	**1.60 (1.22–2.11)**
Resistin (Dyslipidemia) Q2 vs Q1	1.04 (0.67–1.61)	1.25 (0.68–2.31)	1.23 (0.77–1.96)	1.15 (0.86–1.53)
Q3 vs Q1	1.09 (0.69–1.72)	1.36 (0.72–2.57)	0.91 (0.53–1.56)	1.08 (0.80–1.47)
Q4 vs Q1	0.98 (0.62–1.56)	**2.08 (1.21–3.58)**	0.98 (0.59–1.65)	1.21 (0.91–1.61)
Q5 vs Q1	**1.65 (1.09–2.50)**	**1.92 (1.07–3.46)**	1.47 (0.91–2.38)	**1.65 (1.25–2.17)**
Resistin (Medit Diet) Q2 vs Q1	1.08 (0.70–1.66)	1.22 (0.66–2.24)	1.22 (0.76–1.95)	1.15 (0.87–1.53)
Q3 vs Q1	1.06 (0.68–1.67)	1.30 (0.69–2.46)	0.96 (0.56–1.65)	1.08 (0.80–1.46)
Q4 vs Q1	0.96 (0.61–1.52)	**1.92 (1.12–3.32)**	0.97 (0.58–1.63)	1.17 (0.88–1.56)
Q5 vs Q1	**1.62 (1.08–2.45)**	**1.84 (1.02–3.32)**	1.53 (0.95–2.47)	**1.63 (1.24–2.15)**
Resistin (Alcohol) Q2 vs Q1	1.07 (0.70–1.66)	1.23 (0.65–2.30)	1.20 (0.74–1.93)	1.15 (0.86–1.53)
Q3 vs Q1	1.07 (0.68–1.68)	1.30 (0.68–2.46)	0.90 (0.52–1.55)	1.06 (0.78–1.44)
Q4 vs Q1	0.96 (0.60–1.51)	**2.10 (1.21–3.64)**	0.97 (0.58–1.64)	1.19 (0.89–1.59)
Q5 vs Q1	**1.66 (1.10–2.51)**	**1.96 (1.08–3.55)**	1.43 (0.88–2.32)	**1.64 (1.24–2.16)**
Resistin (Social Class) Q2 vs Q1	1.10 (0.70–1.72)	1.20 (0.64–2.26)	1.10 (0.67–1.80)	1.12 (0.84–1.50)
Q3 vs Q1	1.10 (0.69–1.76)	1.30 (0.68–2.52)	0.95 (0.55–1.64)	1.09 (0.80–1.49)
Q4 vs Q1	0.99 (0.62–1.60)	**2.09 (1.18–3.70)**	1.01 (0.59–1.72)	1.21 (0.90–1.64)
Q5 vs Q1	**1.67 (1.08–2.56)**	**1.87 (1.01–3.46)**	1.43 (0.87–2.36)	**1.63 (1.22–2.17)**
Resistin (Smoking) Q2 vs Q1	1.06 (0.69–1.64)	1.18 (0.64–2.17)	1.22 (0.76–1.95)	1.142 (0.86–1.51)
Q3 vs Q1	1.05 (0.67–1.65)	1.27 (0.67–2.39)	0.89 (0.52–1.54)	1.04 (0.77–1.41)
Q4 vs Q1	0.90 (0.57–1.43)	**1.94 (1.12–3.34)**	0.94 (0.56–1.58)	1.13 (0.84–1.50)
Q5 vs Q1	**1.55 (1.03–2.35)**	1.80 (1.00–3.25)	1.38 (0.84–2.24)	**1.55 (1.17–2.05)**
Resistin (Hypertension) Q2 vs Q1	1.02 (0.66–1.58)	1.21 (0.66–2.22)	1.23 (0.77–1.97)	1.13 (0.85–1.50)
Q3 vs Q1	1.04 (0.66–1.64)	1.30 (0.69–2.46)	0.91 (0.53–1.57)	1.05 (0.77–1.42)
Q4 vs Q1	0.96 (0.61–1.52)	**2.04 (1.18–3.53)**	0.99 (0.59–1.66)	1.18 (0.89–1.58)
Q5 vs Q1	**1.63 (1.08–2.46)**	**1.90 (1.06–3.42)**	1.53 (0.95–2.47)	**1.65 (1.26–2.18)**
Resistin (Diabetes) Q2 vs Q1	1.10 (0.71–1.69)	NA	1.28 (0.80–2.04)	1.20 (0.91–1.60)
Q3 vs Q1	1.06 (0.68–1.67)	NA	0.93 (0.54–1.58)	1.06 (0.78–1.43)
Q4 vs Q1	0.98 (0.62–1.56)	NA	1.03 (0.61–1.74)	1.249 (0.94–1.67)
Q5 vs Q1	**1.67 (1.11–2.52)**	NA	1.54 (0.96–2.49)	**1.68 (1.28–2.21)**
Resistin (ACS) Q2 vs Q1	1.08 (0.70–1.66)	1.11 (0.60–2.05)	1.21 (0.75–1.93)	1.13 (0.85–1.50)
Q3 vs Q1	1.08 (0.69–1.69)	1.15 (0.61–2.16)	0.93 (0.55–1.59)	1.05 (0.78–1.42)
Q4 vs Q1	0.96 (0.61–1.52)	**1.96 (1.14–3.38)**	0.98 (0.58–1.64)	1.18 (0.88–1.57)
Q5 vs Q1	**1.67 (1.11–2.52)**	1.64 (0.91–2.95)	1.49 (0.92–2.41)	**1.61 (1.22–2.12)**

*ACS* Acute coronary syndrome, *NA* Not applicable.

Significant values are in bold.

A maximally adjusted model is presented in Table 3 for each cause of mortality, corroborating the effect on all-cause mortality of resistin in quintiles 1.52 (1.13–2.05). The effect of resistin was even higher for cardiovascular deaths (HR = 2.14; 95% CI 1.13–4.06), being exceeded only by suffering diabetes (HR = 3.04; 95% CI 1.98–4.69) or previous acute coronary syndrome (HR = 3.67; 95% CI 2.18–6.18). In addition, resistin was associated with all-cause mortality and cardiovascular mortality, whether it was included as a continuous variable (Supplementary Table S2), or when the treatment with statins, antihypertensives, and antidiabetics was adjusted (Supplementary Table S3).

**Table 3:** The table presents four proportional hazards models, one for each type of mortality. The models adjusted all the variables analyzed and were summarized with the HR (95% CI) among their categories.

	Oncological deaths HR (95% CI)	Cardiovascular deaths HR (95% CI)	Deaths from other causes HR (95% CI)	All-cause deaths HR (95% CI)
Resistin Q2 vs Q1	1.01 (0.63–1.60)	1.32 (0.68–2.54)	1.05 (0.64–1.74)	1.08 (0.80–1.47)
Q3 vs Q1	0.99(0.61–1.60)	1.16 (0.59–2.26	0.81 (0.46–1.42)	0.97 (0.70–1.33)
Q4 vs Q1	0.95 (0.58–1.54)	**2.32 (1.27–4.25)**	0.96 (0.56–1.65)	1.17 (0.86–1.59)
Q5 vs Q1	1.46 (0.94–2.28)	**2.14 (1.13–4.06)**	1.33 (0.80–2.22)	**1.52 (1.13–2.05)**
Age (years)	1.08 (1.06–1.10)	1.11 (1.08–1.14)	1.10 (1.08–1.12)	1.09 (1.08–1.10)
Male sex	2.33 (1.61–3.37)	2.64 (1.60–4.34)	2.77 (1.81–4.23)	2.54 (1.99–3.23)
BMI (kg/m^2^) 25–29 vs < 25	0.58 (0.38–0.87)	1.11 (0.57–2.15)	0.48 (0.30–0.77)	0.60 (0.46–0.79)
≥ 30 vs < 25	0.68 (0.44–1.04)	1.05 (0.53–2.08)	0.60 (0.37–0.97)	0.69 (0.52–0.91)
Physical activity Q2 vs Q1	1.01 (0.65–1.57)	0.75 (0.40–1.41)	0.93 (0.56–1.57)	0.93 (0.69–1.24)
Q3 vs Q1	0.89 (0.55–1.42)	0.78 (0.39–1.53)	0.84 (0.48–1.45)	0.85 (0.62–1.17)
Q4 vs Q1	0.99 (0.65–1.50)	1.07 (0.59–1.93)	0.99 (0.62–1.60)	1.00 (0.75–1.31)
Q5 vs Q1	0.42 (0.23–0.77)	1.18 (0.59–2.37)	0.67 (0.36–1.24)	0.63 (0.44–0.91)
Low HDL cholesterol	1.29 (0.93–1.78)	0.81 (0.52–1.27)	1.26 (0.87–1.83)	1.13 (0.92–1.40)
Mediterranean diet Q2 vs Q1	0.82 (0.51–1.31)	0.93 (0.53–1.62)	0.93 (0.54–1.62)	0.87 (0.65–1.18)
Q3 vs Q1	1.01 (0.65–1.58)	0.74 (0.41–1.34)	01.25 (0.75–2.09)	0.99 (0.74–1.33)
Q4 vs Q1	0.78 (0.47–1.29)	0.71 (0.37–1.37)	1.22 (0.71–2.10)	0.87 (0.63–1.20)
Q5 vs Q1	1.11 (0.68–1.81)	0.43 (0.19–0.97)	0.64 (0.32–1.28)	0.77 (0.54–1.10)
Moderate alcohol vs < 1.5 g/day	0.79 (0.55–1.15)	0.52 (0.31–0.87)	0.86 (0.57–1.29)	0.74 (0.58–0.94)
Excessive alcohol vs < 1.5 g/day	1.00 (0.59–1.67)	0.54 (0.26–1.14)	0.90 (0.49–1.64)	0.82 (0.58–1.16)
Social class Q2 vs Q1	0.83 (0.58–1.19)	1.21 (0.74–1.99)	1.10 (0.71–1.71)	0.99 (0.78–1.26)
Q3 vs Q1	0.55 (0.32–0.93)	1.40 (0.76–2.61)	1.10 (0.63–1.93)	0.88 (0.63–1.21)
Q4 vs Q1	0.70 (0.43–1.14)	0.76 (0.33–1.76)	0.91 (0.50–1.67)	0.80 (0.57–1.12)
Q5 vs Q1	0.39 (0.20–0.75)	0.52 (0.18–1.52)	0.94 (0.50–1.77)	0.59 (0.39–0.89)
Smoking	1.76 (1.24–2.50)	1.79 (1.08–2.98)	1.50 (1.00–2.26)	1.67 (1.32–2.12)
Dyslipidemia	0.89 (0.65–1.22)	1.73 (1.12–2.69)	0.86 (0.60–1.23)	1.02 (0.83–1.26)
Hypertension	1.25 (0.88–1.76)	1.75 (1.06–2.89)	1.11 (0.75–1.64)	1.27 (1.01–1.60)
Diabetes	1.43 (0.99–2.06)	3.04 (1.98–4.69)	2.34 (1.59–3.43)	2.08 (1.67–2.60)
Acute coronary syndrome	0.79 (0.38–1.64)	3.67 (2.18–6.18)	1.56 (0.86–2.85)	1.72 (1.23–2.41)

Resistin significant values are in bold.

To reduce the possible noise from young participants who did not contribute to the outcome, this model was corroborated for cardiovascular and all-cause deaths only in participants older than 39 years at the recruitment, the age above 90% of deaths occurred (Supplementary Table S4). Also, sex-specific models were adjusted for all-cause deaths (Supplementary Table S5). Finally, to reduce any residual confounding attributable to the use of dichotomous variables for several key risk factors we adjusted two more models for all-cause deaths, one with resistin in quintiles and one with resistin in ng/mL (Supplementary Table S6), which include these factors as continuous variables (years smoking, glycaemia, blood pressure, LDL cholesterol, and HDL cholesterol).

**Figure 1:**
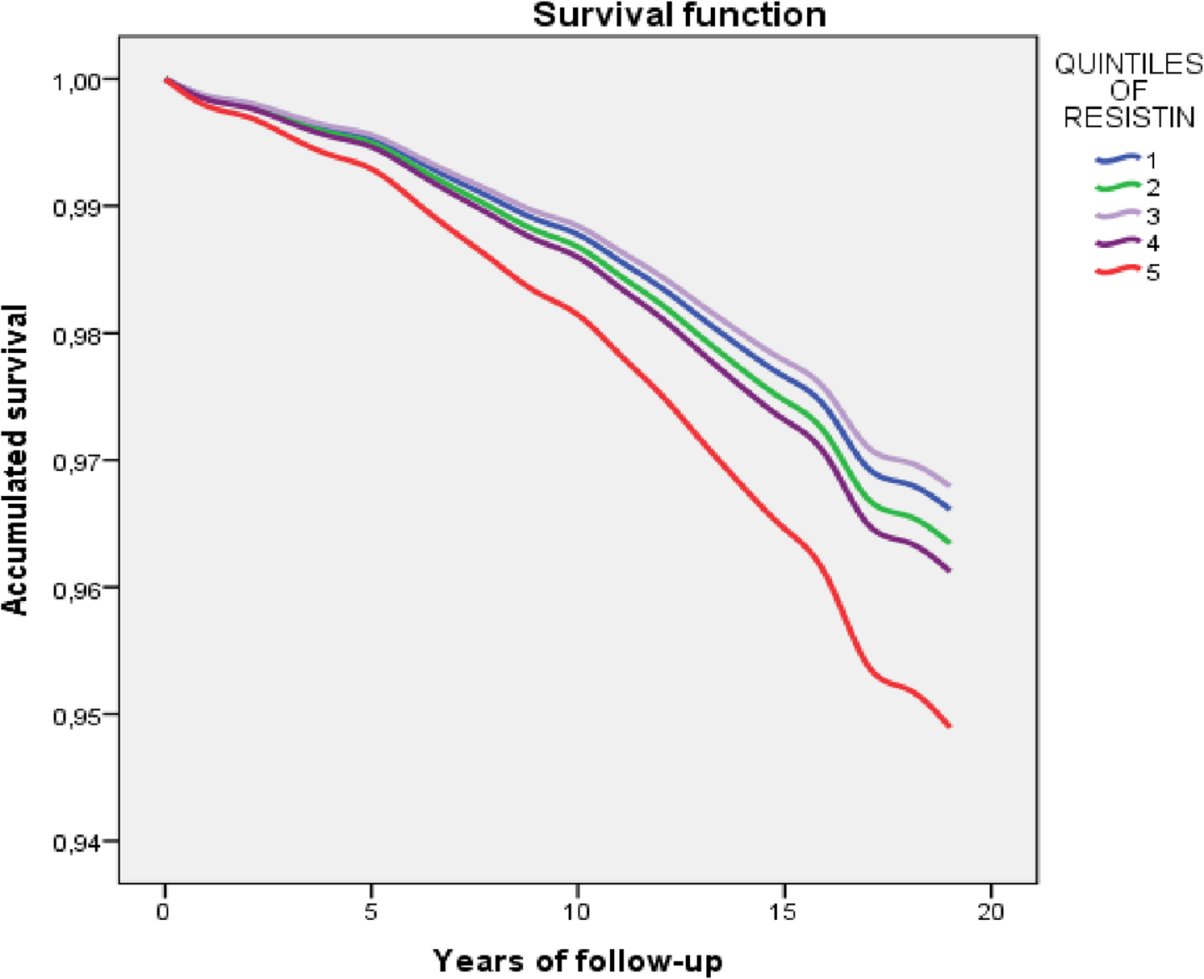
Survival function of the resistin quintiles for all-cause deaths, obtained with the model in Table 3 (n = 6636).

**Figure 2:**
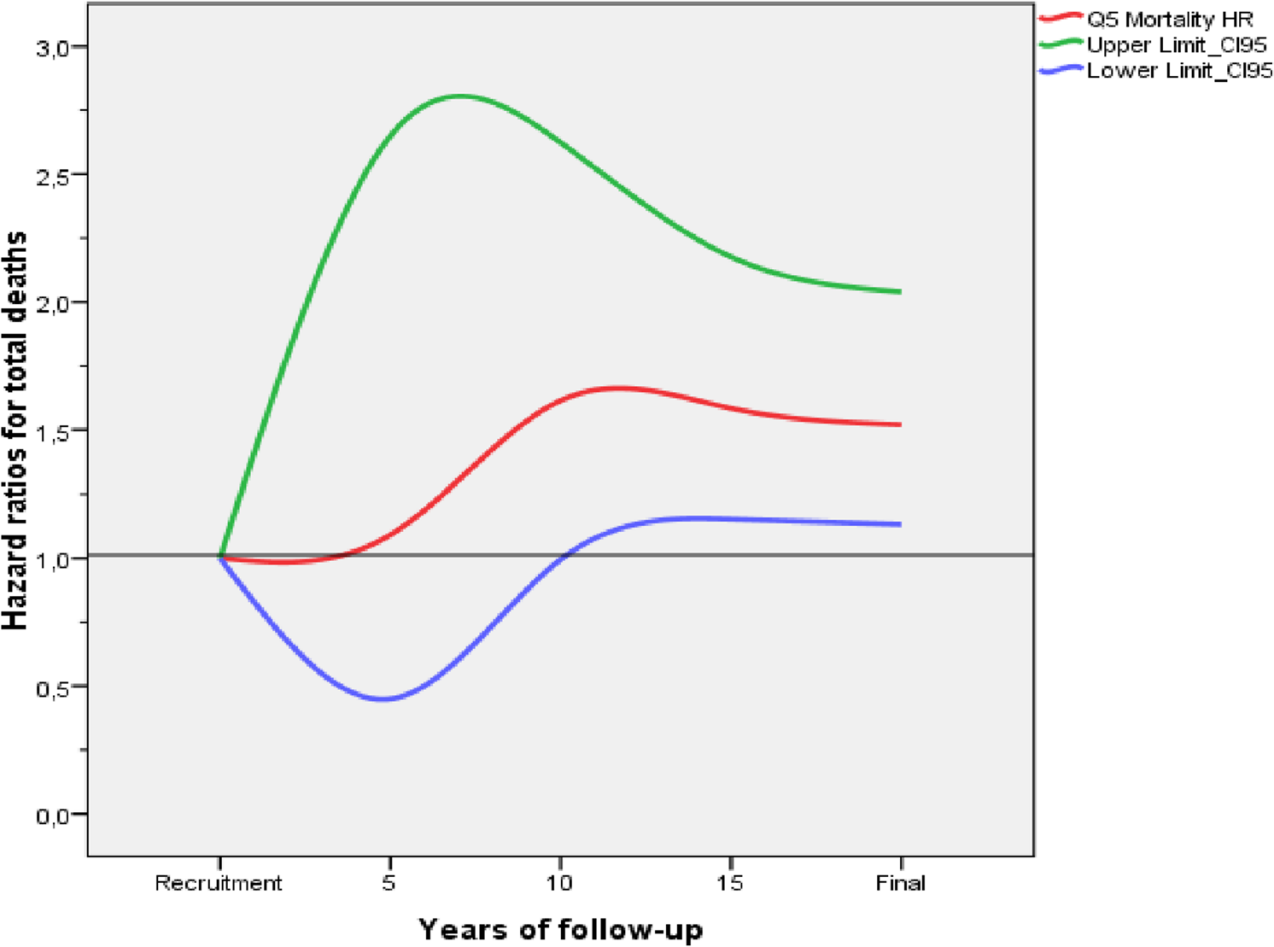
The sequence graph shows the evolution of hazard ratios (95% CI) of resistin Q5 for all-cause deaths at 5, 10 and 15 years of follow-up.

Figure 1 shows the survival function of resistin for the all-cause deaths obtained in the model in Table 3; and Fig. 2 shows the HR (95% CI) of resistin Q5 for all-cause deaths according to years of follow-up, as compared with the risk of resistin Q1.

In the *sensitivity analysis*, the results of resistin were similar in the model where social class was substituted by the participant's educational level (Supplementary Table S7). Results were similar when we used imputed values for missing data of resistin and social class (Supplementary Table S8).

## Discussion

In this study, it was observed that exposure to high serum resistin concentrations was associated with an increased risk of death during follow-up in a general population cohort. Mortality from cardiovascular causes was the one with the strongest association. Adjusting these results for age, sex, and known risk or protective factors against mortality, corroborated the existence of the association between resistin and death regardless of these factors.

To the best of our knowledge, this is the first time that the increased risk of cardiovascular death attributable to resistin has been verified in the general population. Resistin had been pointed out as a risk factor for all-cause mortality in the aged population^9^, but this study included younger people and detected an increase of cardiovascular mortality as an effect of high resistin levels. The inclusion of a younger general population, in which cardiovascular mortality is more relevant than other causes of death, may have helped to detect the risk of cardiovascular death. In addition, the study is an analysis of what is to date the largest population sample with serum resistin measurement, and the cohort has been followed for more than 15 years. Compared with people with low levels of resistin, people with high levels of this cytokine had an increased risk of death of more than 50%, and the risk doubled when mortality from cardiovascular causes was analyzed. These results are consistent with the effect of resistin on the risk of cardiovascular events, which was detected by the authors in this same cohort after the first three and a half years of follow-up^4^, and which other authors have subsequently detected in a multiethnic cohort^18^. It is well known today that resistin is positively associated with serum fibroblast growth factor 23, which is associated with cardiovascular disease and all-cause mortality in patients with diabetes mellitus^19^. For all the above reasons, it has been suggested that resistin could have diagnostic utility or even be a target of therapies against cardiovascular disease^20^.

However, to date the number of participants and the follow-up time of published studies has been insufficient to detect the effect of resistin on cardiovascular mortality. This had only been detected by studying population groups at high risk of death, either through meta-analysis of patients with cardiovascular diseases over 60 years of age^21^, either by following a cohort of people over 70 years of age^9^, or in patients with diabetes over 60 years of age years^22^. This is the first study to demonstrate the effect of resistin on cardiovascular mortality in the general population.

Among the other traditional determinants of death analyzed here, the results were as expected for the increase in risk with age, male sex, smoking, hypertension, diabetes and coronary syndrome. And equally expected was the lower risk of dying among those who were in the highest quintiles of social class, whose effect cannot be explained by lifestyle factors such as smoking, diet or physical activity^23^. The protective effect found for moderate consumption of alcohol in the general population has been previously described^24^ and is controversial^25^. On the other hand, for being overweight and obesity, there was the paradoxical protective association with mortality that has been described previously^26^, and the controversy surrounding it has been debated^27^. In the present study, no effect of being overweight and obesity on mortality was detected when BMI and the remaining risk factors were adjusted as continuous variables. None of the traditional risk factors modified the effect of resistin on mortality when they were included in the models.

Among the potential limitations of the study are the attribution of causes of death; these are based on medical death certificates, which should be taken with caution even though the accuracy of the certificates has been found to be acceptable for epidemiological studies^28^. Added to this is the authors’ decision to include deaths attributed to diabetes as a cardiovascular cause. However, the above does not affect the identification of each deceased person in Spain, which is a rigorous process and guarantees the accounting of all-cause deaths, which are the main result of the study. A second limitation is that only resistin measurements at recruitment are analyzed, so their values will have undergone variations during follow-up. However, the results of the present study are consistent with those obtained with only three and a half years of follow-up for the relationship between resistin and acute coronary syndrome^4^, and corroborate the previous findings in populations at high risk of death in the general population^9,21,22^. Finally, the authors think that the exclusion of the ninety-three participants (1.4% of the cohort) in whom resistin could not be determined does not introduce bias into the study because the absence was due, in all cases, to problems of hemolysis in some serum samples or the impossibility of extracting blood; in addition, the imputation of values for missing data did not change the results.

The main strength of the study lies in the fact that it analyzed serum resistin in the largest population sample measured to date, with a wide range of ages, with follow-up for more than fifteen years, and adjusted for its effect on mortality due to traditional risk factors. This has allowed the verification of the effect of resistin on mortality in the general population.

In conclusion, people in the general population exposed to high levels of serum resistin have a higher risk of death than those with lower levels of this cytokine, mainly due to cardiovascular mortality.

## Supplementary Information

Supplementary Tables.

## Data Availability

The data, the Bioethics Committee approval and the analysis plan that support the findings of this study are available on request from the corresponding author.

## References

[1] Steppan CM, Bailey ST, Bhat S (2001). The hormone resistin links obesity to diabetes. Nature.

[2] McTernan CL, McTernan PG, Harte AL, Levick PL, Barnett AH, Kumar S (2002). Resistin, central obesity, and type 2 diabetes. Lancet.

[3] Patel L, Buckels AC, Kinghorn IJ (2003). Resistin is expressed in human macrophages and directly regulated by PPAR gamma activators. Biochem. Biophys. Res. Commun..

[4] Cabrera de León A, Almeida González D, González Hernández A (2014). The association of resistin with coronary disease in the general population. J. Atheroscler. Thromb..

[5] Cabrera de León A, Almeida González D, González Hernández A (2014). Relationships between serum resistin and fat intake, serum lipid concentrations and adiposity in the general population. J. Atheroscler. Thromb..

[6] Marcelino-Rodríguez I, Almeida González D, Alemán-Sánchez JJ (2017). Inverse association of resistin with physical activity in the general population. PLoS ONE.

[7] Zhang JZ, Gao Y, Zheng YY (2017). Increased serum resistin level is associated with coronary heart disease. Oncotarget.

[8] Su KZ, Li YR, Zhang D (2019). Relation of circulating resistin to insulin resistance in type 2 diabetes and obesity, a systematic review and meta-analysis. Front. Physiol..

[9] Parkkila K, KiviniemiI A, Tulppo M (2021). Resistin is a risk factor for all-cause mortality in elderly Finnish population: A prospective study in the OPERA cohort. PLoS ONE.

[10] Scarale MG, Antonucci A, Cardellini M (2021). A serum resistin and multi-cytokine inflammatory pathway is linked with and helps predict all-cause death in diabetes. J. Clin. Endocrinol. Metab..

[11] Cabrera de León A, Rodríguez Pérez MC, Almeida González D (2008). Presentation of the “CDC de Canarias” cohort: Objectives, design and preliminary results. Rev. Esp. Salud. Publica..

[12] Aguirre-Jaime A, Cabrera de León A, Domínguez Coello S (2008). Validation of a food intake frequency questionnaire adapted for the study and monitoring of the adult population of the Canary Islands, Spain. Rev. Esp. Salud. Publica.

[13] Trichopoulou A, Costacou T, Bamia C, Trichopoulos D (2003). Adherence to a mediterranean diet and survival in a Greek population. N. Engl. J. Med..

[14] Elosua R, García M, Aguilar A, Molina L, Covas MI, Marrugat J (2000). On behalf of Investigators of the MARATDON Group. Validation of the Minnesota leisure time physical activity questionnaire in Spanish women. Med. Sci. Sports Exerc..

[15] Ainsworth BE, Haskell WL, Herrmann SD (2011). 2011 Compendium of Physical Activities: A second update of codes and MET values. Med. Sci. Sports Exerc.

[16] Bernstein MS, Morabia A, Sloutskis D (1999). Definition and prevalence of sedentarism in an urban population. Am. J. Public Health.

[17] Cabrera de León A, Rodríguez Pérez MC, Domínguez Coello S (2009). Validation of the ICE model to assess social class in the adult population. Rev. Esp. Salud. Publica.

[18] Muse ED, Feldman DI, Blaha MJ (2015). The association of resistin with cardiovascular disease in the Multi-Ethnic Study of Atherosclerosis. Atherosclerosis.

[19] Nakashima A, Yokoyama K, Kawanami D (2018). Association between resistin and fibroblast growth factor 23 in patients with type 2 diabetes mellitus. Sci. Rep..

[20] Zhou L, Li JY, He PP, Yu XH, Tang CK (2021). Resistin: Potential biomarker and therapeutic target in atherosclerosis. Clin. Chim. Acta.

[21] Fontana A, Spadaro S, Copetti M (2015). Association between resistin levels and all-cause and cardiovascular mortality: A new study and a systematic review and meta-analysis. PLoS ONE.

[22] Fontana A, Ortega Moreno L, Lamacchia O (2017). Serum resistin is causally related to mortality risk in patients with type 2 diabetes: Preliminary evidences from genetic data. Sci. Rep..

[23] Zhang YB, Chen C, Pan XF (2021). Associations of healthy lifestyle and socioeconomic status with mortality and incident cardiovascular disease: Two prospective cohort studies. BMJ.

[24] Bo X, Sreenivas PV, Min Z, Chuanwei M, Yinkun Y, Jie M (2017). Relationship of alcohol consumption to all-cause, cardiovascular, and cancer-related mortality in US adults. J. Am. Coll. Cardiol.

[25] Barbería-Latasa M, Gea A, Martínez-González MA (2022). Alcohol, drinking pattern, and chronic disease. Nutrients.

[26] Flegal KM, Kit BK, Orpana H, Graubard BI (2013). Association of all-cause mortality with overweight and obesity using standard body mass index categories: A systematic review and meta-analysis. JAMA.

[27] Berrigan D, Troiano RP, Graubard BI (2016). BMI and mortality: The limits of epidemiological evidence. Lancet.

[28] Eriksson A, Stenlund H, Ahlm K (2013). Accuracy of death certificates of cardiovascular disease in a community intervention in Sweden. Scand. J. Public Health.

