# Supplementary material for: Resistin as a risk factor for all-cause (and cardiovascular) death in the general population
Source: Sci Rep. 2022 Nov 15;12:19627. doi: 10.1038/s41598-022-24039-2 (PMC9666658; doi:10.1038/s41598-022-24039-2)
Supplement: Supplementary file 1 — Supplementary Tables. [file 41598_2022_24039_MOESM1_ESM.docx]

**Resistin as a risk factor for all-cause (and cardiovascular) death in the general population.**

**Authors**: María del Cristo Rodríguez Pérez, Delia Almeida González, Itahisa Marcelino Rodríguez, Santiago Domínguez Coello, Francisco J Cuevas Fernández, Buenaventura Brito Díaz, and Antonio Cabrera de León.

**Table of contents**:

Table S1. Association between exposure factors and mortality. Four Cox models adjusted for age and sex are presented for each factor, summarized with the HR (95% CI). Page 2

Table S2. The table presents four proportional hazards models, one for each type of mortality. The models adjusted all the variables analyzed and were summarized with the HR (95% CI). Resistin was included as a continuous variable Page 3

Table S3. The table presents four proportional hazards models, one for each type of mortality, adjusting all the variables analyzed plus the treatment with statins, antihypertensives, and antidiabetics. Page 4

Table S4. The table presents two proportional hazards models, one for each type of mortality, in participants older than 39 years at the recruitment. The models adjusted all the variables analyzed and were summarized with the HR (95% CI). Page 5

Table S5. The table presents two proportional hazards models for the all-cause deaths, separately in women and men. The models adjusted all the variables analyzed and were summarized with the HR (95% CI). Page 6

Table S6. The table presents two proportional hazard models for all-cause deaths, one with resistin in quintiles and one with resistin in ng/mL, which substitute some risk factors as smoking, diabetes, hypertension and dyslipidemia for continuous variables (years smoking, glycaemia, blood pressure, LDL cholesterol, and HDL cholesterol). Page 7

Table S7. The table presents a proportional hazards model for all-cause mortality, with social class being substituted by the participant's educational level. Page 8

Table S1. Association between exposure factors and mortality. Four Cox models adjusted for age and sex are presented for each factor, summarized with the HR (95% CI).

| **Exposure factors** | Oncological  deaths  HR (95% CI) | Cardiovascular deaths  HR (95% CI) | Deaths from other causes  HR (95% CI) | All-cause  Deaths  HR (95% CI) |
| --- | --- | --- | --- | --- |
| Resistin Q2 vs Q1 | 1.07 (0.70-1.65) | 1.39 (0.70-2.78) | 1.15 (0.74-1.79) | 1.15 (0.87-1.53) |
| Q3 vs Q1 | 1.04 (0.66-1.64) | 1.45 (0.71-2.99) | 0.95 (0.58-1.56) | 1.07 (0.79-1.44) |
| Q4 vs Q1 | 0.96 (0.61-1.52) | **2.32 (1.24-4.31)** | 1.03 (0.64-1.65) | 1.19 (0.89-1.58) |
| Q5 vs Q1 | **1.66 (1.10-2.49)** | **2.06 (1.05-4.04)** | 1.50 (0.96-2.34) | **1.67 (1.23-2.18)** |
| Age (years)^1^ | 1.09 (1.07-1.10) | 1.13 (1.10-1.16) | 1.11 (1.09-1.13) | 1.10 (1.09-1.11) |
| Male sex^2^ | 1.97 (1.48-2.60) | 2.26 (1.47-3.50) | 2.38 (1.74-3.26) | 2.17 (1.80-2.63) |
| BMI (Kg/m^2^) 25- 29 vs <25 | 0.57 (0.39-0.83) | 1.07 (0.55-2.06) | 0.57 (0.38-0.86) | 0.63 (0.49-0.81) |
| ≥30 vs <25 | 0.77 (0.53-1.11) | 1.38 (0.72-2.67) | 0.82 (0.55-1.23) | 0.86 (0.67-1.10) |
| Physical activity Q2 vs Q1 | 0.99 (0.66-1.49) | 0.87 (0.47-1.62) | 0.82 (0.51-1.30) | 0.90 (0.69-1.19) |
| Q3 vs Q1 | 0.76 (0.48-1.19) | 0.71 (0.35-1.42) | 0.85 (0.53-1.34) | 0.78 (0.58-1.05) |
| Q4 vs Q1 | 0.91 (0.62-1.35) | 0.75 (0.41-1.38) | 0.83 (0.55-1.27) | 0.85 (0.66-1.10) |
| Q5 vs Q1 | 0.52 (0.30-0.88) | 0.87 (0.43-1.75) | 0.56 (0.32-0.98) | 0.59 (0.42-0.83) |
| Low HDL cholesterol | 1.43 (1.07-1.91) | 1.18 (0.76-1.84) | 1.41 (1.03-1.92) | 1.37 (1.13-1.66) |
| Mediterranean diet Q2 vs Q1 | 0.72 (0.46-1.12) | 0.68 (0.38-1.22) | 0.80 (0.51-1.26) | 0.74 (0.56-0.98) |
| Q3 vs Q1 | 0.96 (0.64-1.44) | 0.69 (0.39-1.22) | 0.88 (0.57-1.36) | 0.86 (0.66-1.12) |
| Q4 vs Q1 | 0.69 (0.43-1.10) | 0.54 (0.29-1.03) | 0.78 (0.48-1.24) | 0.68 (0.51-0.92) |
| Q5 vs Q1 | 0.95 (0.60-1.50) | 0.19 (0.06-0.53) | 0.53 (0.30-0.93) | 0.61 (0.44-0.84) |
| Moderate alcohol vs <1.5 g/d | 0.81 (0.58-1.14) | 0.45 (0.27-0.76) | 0.70 (0.49-1.00) | 0.68 (0.55-0.85) |
| Excessive alcohol vs <1.5 g/d | 0.96 (0.59-1.55) | 0.51 (0.24-1.08) | 0.66 (0.39-1.13) | 0.73 (0.53-1.01) |
| Social class Q2 vs Q1 | 0.84 (0.59-1.19) | 0.95 (0.55-1.62) | 1.28 (0.86-1.91) | 1.00 (0.79-1.27) |
| Q3 vs Q1 | 0.50 (0.30-0.85) | 1.13 (0.58-2.18) | 1.09 (0.65-1.83) | 0.80 (0.58-1.10) |
| Q4 vs Q1 | 0.72 (0.45-1.16) | 0.52 (0.21-1.26) | 0.87 (0.49-1.53) | 0.74 (0.53-1.03) |
| Q5 vs Q1 | 0.46 (0.25-0.85) | 0.47 (0.16-1.35) | 0.96 (0.52-1.77) | 0.62 (0.41-0.92) |
| Smoking | 1.99 (1.45-2.72) | 1.42 (0.85-2.38) | 1.61 (1.12-2.29) | 1.73 (1.40-2.14) |
| Dyslipidemia | 0.81 (0.60-1.09) | 2.01 (1.35-3.01) | 0.89 (0.64-1.24) | 1.04 (0.86-1.26) |
| Hypertension | 1.13 (0.83-1.54) | 1.73 (1.06-2.83) | 1.16 (0.84-1.61) | 1.23 (1.01-1.51) |
| Diabetes | 1.53 (1.10-2.14) | 2.57 (1.65-4.00) | 2.92 (2.12-4.01) | 2.20 (1.80-2.70) |
| Acute coronary syndrome | 0.84 (0.41-1.71) | 3.89 (2.23-6.73) | 2.22 (1.37-3.60) | 1.93 (1.41-2.66) |

^1^Adjusted for sex only. ^2^Adjusted for age only.

Table S2. The table presents four proportional hazards models, one for each type of mortality, with resistin included as a continuous variable. The models adjusted all the variables analyzed and were summarized with the HR (95% CI).

|  | Oncological  deaths  HR (95% CI) | Cardiovascular deaths  HR (95% CI) | Deaths from other causes  HR (95% CI) | All-cause  deaths  HR (95% CI) |
| --- | --- | --- | --- | --- |
| Resistin (ng/mL) | 1.05 (0,99-1.11) | **1.09 (1.01-1.16)*** | 1.02 (0.95-1.10) | **1.05 (1.01-1.09)*** |
| Age (years) | 1.08 (1.06-1.10) | 1.11 (1.08-1.14) | 1.10 (1.08-1.12) | 1.09 (1.08-1.10) |
| Male sex | 2.33 (1.61-3.37) | 2.60 (1.58-4.28) | 2.75 (1.80-3.19) | 2.53 (1.98-3.22) |
| BMI (Kg/m^2^) 25- 29 vs <25 | 0.58 (0.39-0.87) | 1.06 (0.54-2.06) | 0.49 (0.31-0.87) | 0.60 (0.46-0.79) |
| ≥30 vs <25 | 0.67 (0.44-1.04) | 1.06 (0.53-2.09) | 0.60 (0.37-0.97) | 0.69 (0.52-0.91) |
| Physical activity Q2 vs Q1 | 1.01 (0.65-1.57) | 0.75 (0.40-1.41) | 0.94 (0.56-1.60) | 0.93 (0.69-1.25) |
| Q3 vs Q1 | 0.90 (0,56-1.44) | 0.72 (0.36-1.42) | 0.85 (0,49-1.48) | 0.85 (0.62-1.16) |
| Q4 vs Q1 | 1.00 (0,66-1.52) | 1.04 (0.57-1.88) | 1.01 (0.63-1.62) | 1.00 (0.76-1.32) |
| Q5 vs Q1 | 0.43 (0.23-0.79) | 1.16 (0.58-2.34) | 0.68 (0.37-1.25) | 0.64 (0.44-0.92) |
| Low HDL cholesterol | 1.30 (0.94-1.80) | 0.84 (0.54-1.30) | 1.28 (0.89-1.85) | 1.15 (0.93-1.42) |
| Mediterranean diet Q2 vs Q1 | 0.82 (0.51-1.31) | 0.95 (0.55-1.67) | 0.92 (0.53-1.60) | 0.87 (0.64-1.17) |
| Q3 vs Q1 | 1.01 (0.65-1.57) | 0.75 (0.41-1.35) | 1.25 (0.75-2.09) | 0.99 (0.74-1.32) |
| Q4 vs Q1 | 0.77 (0.47-1.28) | 0.73 (0.38-1.40) | 1.21 (0.70-2.08) | 0.87 (0.63-1.20) |
| Q5 vs Q1 | 1.11 (0.68-1.82) | 0.45 (0.20-1.01) | 0.64 (0.32-1.28) | 0.77 (0.54-1.10) |
| Moderate alcohol vs <1.5 g/d | 0.80 (0.55-1.15) | 0.54 (0.32-0.91) | 0.88 (0.58-1.32) | 0.75 (0.59-0.95) |
| Excessive alcohol vs <1.5 g/d | 1.00 (0.60-1.68) | 0.55 (0.26-1.16) | 0.91 (0.50-1.66) | 0.83 (0.59-1.17) |
| Social class Q2 vs Q1 | 0.83 (0.58-1.20) | 1.15 (0.70-1.88) | 1.11 (0.71-1.72) | 0.99 (0.78-1.26) |
| Q3 vs Q1 | 0.55 (0.32-0.94) | 1.33 (0.72-2.47) | 1.11 (0.64-1.94) | 0.88 (0.63-1.21) |
| Q4 vs Q1 | 0.70 (0.43-1.14) | 0.76 (0.33-1.77) | 0.92 (0.51-1.69) | 0.80 (0.57-1.13) |
| Q5 vs Q1 | 0.39 (0.20-0.75) | 0.54 (0.19-1.57) | 0.95 (0.50-1.79) | 0.60 (0.39-0.90) |
| Smoking | 1.75 (1.24-2.49) | 1.80 (1.08-2.98) | 1.50 (0.99-2.26) | 1.68 (1.33-2.12) |
| Dyslipidemia | 0.89 (0.65-1.23) | 1.80 (1.16-2.79) | 0.86 (0.60-1.24) | 1.03 (0.84-1.27) |
| Hypertension | 1.25 (0.89-1.76) | 1.74 (1.05-2.90) | 1.10 (0.75-1.63) | 1.27(1.02-1.60) |
| Diabetes | 1.43 (0.99-2.06) | 2.85 (1.85-4.37) | 2.31 (1.57-3.39) | 2.05 (1.64-2.56) |
| Acute coronary syndrome | 0.78 (0.38-1.62) | 3.46 (2.04-5.86) | 1.56 (0.86-2.85) | 1.69 (1.21-2.36) |

* When resistin was transformed by taking its squared root, its result was **1.26 (1.02-1.56)** for the all-cause deaths and **1.66 (1.12-2.46)** for the cardiovascular deaths.

Table S3. The table presents four proportional hazards models, one for each type of mortality, adjusting all the variables analyzed plus the treatment with statins, antihypertensives, and antidiabetics.

|  | Oncological  deaths  HR (95% CI) | Cardiovascular deaths  HR (95% CI) | Deaths from other causes  HR (95% CI) | All-cause  deaths  HR (95% CI) |
| --- | --- | --- | --- | --- |
| Resistin Q2 vs Q1 | 1.01 (0.64-1.61) | 1.20 (0.62-2.33) | 1.06 (0.64-1.75) | 1.06 (0.79-1.44) |
| Q3 vs Q1 | 0.99 (0.61-1.61) | 1.08 (0.55-2.13) | 0.80 (0.45-1.40) | 0.94 (0.69-1.30) |
| Q4 vs Q1 | 0.95 (0.58-2.28) | **2.21 (1.20-4.04)** | 0.95 (0.55-1.62) | 1.16 (0.83-1.53) |
| Q5 vs Q1 | 1.46 (0.94-2.28) | **1.96 (1.03-3.74)** | 1.33 (0.80-2.22) | **1.51 (1.12-2.02)** |
| Age (years) | 1.08 (1.06-1.10)   \| 1,082 \| 1,064 \| 1,100 \| \| --- \| --- \| --- \|  \| 1,082 \| 1,064 \| 1,100 \| \| --- \| --- \| --- \|   1,100 | 1.11 (1.08-1.14) | 1.10 (1.08-1.12) | 1.09 (1.08-1.10) |
| Male sex | 2.33 (1.61-3.37) | 2.49 (1.50-4.13) | 2.72 (1.78-4.16) | 2.50 (1.96-3.19) |
| BMI (Kg/m2) 25- 29 vs <25 | 0.58 (0.38-0.87) | 1.15 (0.59-2.25) | 0.48 (0.30-0.77) | 0.61 (0.46-0.80) |
| ≥30 vs <25 | 0.68 (0.44-1.10) | 1.06 (0.53-2.12) | 0.59 (0.36-0.95) | 0.69 (0.52-0.93) |
| Physical activity Q2 vs Q1 | 1.01 (0.65-1.57) | 0.80 (0.43-1.50) | 0.91 (0.54-1.53) | 0.94 (0.70-1.27) |
| Q3 vs Q1 | 0.90 (0.55-1.43) | 0.83 (0.42-1.64) | 0.84 (0.49-1.46) | 0.84 (0.61-1.15) |
| Q4 vs Q1 | 1.00 (0.66-1.52) | 1.07 (0.59-1.94) | 0.97 (0.60-1.56) | 0.97 (0.74-1.28) |
| Q5 vs Q1 | 0.42 (0.23-0.77) | 1.21 (0.60-2.43) | 0.67 (0.36-1.24) | 0.62 (0.43-0.89) |
| Low HDL cholesterol | 1.29 (0.93-1.78) | 0.85 (0.54-1.32) | 1.25 (0.87-1.82) | 1.13 (0.91-1.40) |
| Mediterranean diet Q2 vs Q1 | 0.82 (0.51-1.32) | 0.96 (0.55-1.68) | 0.93 (0.54-1.62) | 0.89 (0.66-1.21) |
| Q3 vs Q1 | 1.01 (0.65-1.60) | 0.79 (0.44-1.41) | 1.25 (0.74-2.10) | 1.04 (0.78-1.39) |
| Q4 vs Q1 | 0.77 (0.46-1.28) | 0.79 (0.41-1.53) | 1.20 (0.69-2.07) | 0.92 (0.67-1.26) |
| Q5 vs Q1 | 1.29 (0.93-1.78) | 0.48 (0.21-1.09) | 0.64 (0.32-1.28) | 0.80 (0.56-1.14) |
| Moderate alcohol vs <1.5 g/d | 0.79 (0.55-1.15) | 0.55 (0.32-0.92) | 0.85 (0.57-1.28) | 0.73 (0.57-0.92) |
| Excessive alcohol vs <1.5 g/d | 1.00 (0.59-1.66) | 0.59 (0.28-1.24) | 0.89 (0.48-1.63) | 0.83 (0.59-1.17) |
| Social class Q2 vs Q1 | 0.83 (0.57-1.19) | 1.18 (0.72-1.94) | 1.11 (0.71-1.72) | 0.99 (0.77-1.26) |
| Q3 vs Q1 | 0.55 (0.32-0.93) | 1.36 (0.73-2.54) | 1.12 (0.64-1.95) | 0.87 (0.63-1.20) |
| Q4 vs Q1 | 0.71 (0.44-1.14) | 0.75 (0.32-1.75) | 0.91 (0.50-1.65) | 0.79 (0.56-1.12) |
| Q5 vs Q1 | 0.39 (0.20-0.75) | 0.49 (0.17-1.42) | 0.91 (0.48-1.72) | 0.58 (0.38-0.87) |
| Smoking | 1.76 (1.24-2.50) | 1.74 (1.05-2.89) | 1.50 (1.00-2.27) | 1.67 (1.32-2.11) |
| Dyslipidemia | 0.87 (0.59-1.28) | 2.00 (1.20-3.33) | 1.03 (0.68-1.56) | 0.94 (0.73-1.21) |
| Hypertension | 1.29 (0.88-2.00) | 1.65 (0.94-2.90) | 1.04 (0.67-1.64) | 1.25 (0.97-1.62) |
| Diabetes | 1.46 (0.93-2.30) | 1.81 (0.98-3.33) | 2.44 (1.55-3.84) | 1.87 (1.41-2.47) |
| Acute coronary syndrome | 0.79 (0.38-1.65) | 3.82 (2.23-6.54) | 1.65 (0.90-3.03) | 1.68 (1.19-2.36) |
| Statins | 0.93 (0.57-1.52) | 1.34 (0.78-2.30) | 1.60 (0.92-2.79) | 1.06 (0.78-1.45) |
| Antihypertensives | 0.92 (0.61-1.38) | 1.07 (0.65-1.78) | 1.18 (0.74-1.88) | 1.06 (0.82-1.38) |
| Antidiabetics | 1.05 (0.57-1.95) | 0.40 (0.21-0.76) | 1.11 (0.61-2.00) | 0.79 (0.56-1.11) |

Table S4. The table presents two proportional hazards models, one for cardiovascular mortality and one for all-cause mortality, in participants older than 39 years at the recruitment. The models adjusted all the variables analyzed and were summarized with the HR (95% CI).

|  | Cardiovascular deaths  HR (95% CI) | All-cause  deaths  HR (95% CI) |
| --- | --- | --- |
| Resistin Q2 vs Q1 | 1,43 (0,74-2,78) | 1,09 (0,80-1,50) |
| Q3 vs Q1 | 1,22 (0,62-2,40) | 1,07 (0,78-1,48) |
| Q4 vs Q1 | **2,36 (1,27-4,39)** | 1,15 (0,83-1,58) |
| Q5 vs Q1 | **2,03 (1,04-3,96)** | **1,45 (1,00-1,99)** |
| Age (years) | 1,11 (1,07-1,15) | 1,09 (1,08-1,11) |
| Male sex | 2,77 (1,67-4,60) | 2,66 (2,06-3,44) |
| BMI (Kg/m^2^) 25- 29 vs <25 | 1,13 (0,57-2,24) | 0,62 (0,46-0,83) |
| ≥30 vs <25 | 1,07 (0,53-2,18) | 0,67 (0,50-0,91) |
| Physical activity Q2 vs Q1 | 0,76 (0,41-1,43) | 0,95 (0,70-10,30) |
| Q3 vs Q1 | 0,71 (0,35-1,44) | 0,92 (0,67-10,27) |
| Q4 vs Q1 | 1,00 (0,54-1,84) | 0,97 (0,72-10,30) |
| Q5 vs Q1 | 1,06 (0,50-2,25) | 0,63 (0,42-0,93) |
| Low HDL cholesterol | 0,81 (0,51-1,27) | 1,13 (0,90-1,41) |
| Mediterranean diet Q2 vs Q1 | 0,93 (0,53-1,63) | 0,91 (0,66-1,24) |
| Q3 vs Q1 | 0,75 (0,41-1,37) | 1,00 (0,74-1,36) |
| Q4 vs Q1 | 0,70 (0,36-1,37) | 0,80 (0,57-1,20) |
| Q5 vs Q1 | 0,48 (0,21-1,08) | 0,78 (0,54-1,13) |
| Moderate alcohol vs <1.5 g/d | 0,50 (0,30-0,86) | 0,67 (0,52-0,87) |
| Excessive alcohol vs <1.5 g/d | 0,37 (0,16-0,87) | 0,74 (0,51-1,06) |
| Social class Q2 vs Q1 | 1,20 (0,73-1,98) | 1,01 (0,79-1,30) |
| Q3 vs Q1 | 1,27 (0,67-2,42) | 0,86 (0,61-1,21) |
| Q4 vs Q1 | 0,58 (0,22-1,52) | 0,83 (0,57-1,20) |
| Q5 vs Q1 | 0,56 (0,19-1,63) | 0,62 (0,40-0,98) |
| Smoking | 1,70 (1,00-2,90) | 1,57 (1,22-2,02) |
| Dyslipidemia | 1,71 (1,09-2,68) | 1,01 (0,82-1,25) |
| Hypertension | 1,48 (0,90-2,43) | 1,25 (0,99-1,58) |
| Diabetes | 2,99 (1,93-4,63) | 2,06 (1,65-2,59) |
| Acute coronary syndrome | 3,65 (2,16-6,17) | 1,72 (1,23-2,41) |

Table S5. The table presents two proportional hazards models for the all-cause deaths, separately in women and men. The models adjusted all the variables analyzed and were summarized with the HR (95% CI).

|  | All-cause  deaths in women  HR (95% CI) | All-cause  deaths in men  HR (95% CI) |
| --- | --- | --- |
| Resistin Q2 vs Q1 | 1,21 (0,74-1,98) | 0,95 (0,641,40) |
| Q3 vs Q1 | 1,00 (0,58-1,72) | 0,98 (0,66-1,46) |
| Q4 vs Q1 | 1,05 (0,62-1,77) | 1,23 (0,84-1,80) |
| Q5 vs Q1 | **1,67 (1,02-2,74)** | **1,50 (1,03-2,19)** |
| Age (years) | 1,12 (1,09-1,14) | 1,08 (1,07-1,10) |
| BMI (Kg/m^2^) 25- 29 vs <25 | 0,73 (0,46-1,17) | 0,56 (0,40-0,79) |
| ≥30 vs <25 | 0,87 (0,54-1,40) | 0,65 (0,45-0,94) |
| Physical activity Q2 vs Q1 | 1,11 (0,74-1,66) | 0,76 (0,49-1,19) |
| Q3 vs Q1 | 0,71 (0,42-1,20) | 0,98 (0,65-1,47) |
| Q4 vs Q1 | 1,08 (0,66-1,76) | 1,03 (0,73-1,45) |
| Q5 vs Q1 | 0,44 (0,11-1,82) | 0,61 (0,41-0,91) |
| Low HDL cholesterol | 1,37 (0,98-1,91) | 0,96 (0,72-1,28) |
| Mediterranean diet Q2 vs Q1 | 1,29 (0,80-2,07) | 0,64 (0,43-0,95) |
| Q3 vs Q1 | 1,44 (0,90-2,28) | 0,75 (0,51-1,10) |
| Q4 vs Q1 | 1,29 (0,75-2,23) | 0,71 (0,48-1,05) |
| Q5 vs Q1 | 0,60 (0,28-1,27) | 0,74 (0,49-1,12) |
| Moderate alcohol vs <1.5 g/d | 0,84 (0,55-1,28) | 0,66 (0,50-0,89) |
| Excessive alcohol vs <1.5 g/d | 0,00 (0,00-3,42) | 0,79 (0,55-1,13) |
| Social class Q2 vs Q1 | 1,03 (0,69-1,54) | 1,00 (0,73-1,36) |
| Q3 vs Q1 | 0,71 (0,42-1,20) | 1,08 (0,71-1,63) |
| Q4 vs Q1 | 0,62 (0,32-1,19) | 0,91 (0,60-1,36) |
| Q5 vs Q1 | 1,06 (0,55-2,06) | 0,43 (0,25-0,76) |
| Smoking | 2,42 (1,49-3,92) | 1,53 (1,16-2,00) |
| Dyslipidemia | 1,09 (0,77-1,54) | 0,93 (0,72-1,21) |
| Hypertension | 1,11 (0,76-1,62) | 1,35 (1,01-1,80) |
| Diabetes | 2,33 (1,63-3,34) | 2,06 (1,55-2,75) |
| Acute coronary syndrome | 1,88 (1,06-3,33) | 1,70 (1,12-2,59) |

Table S6. The table presents two proportional hazard models for all-cause deaths, one with resistin in quintiles and one with resistin in ng/mL, which substitute some risk factors as smoking, diabetes, hypertension and dyslipidemia for continuous variables (years smoking, glycaemia, blood pressure, LDL cholesterol, and HDL cholesterol).

|  | All-cause deaths  HR (95% CI) |  | All-cause deaths  HR (95% CI) |
| --- | --- | --- | --- |
| Resistin Q2 vs Q1 | 1.02 (0.76-1.38) | Resistin (ng/mL) |  |
| Q3 vs Q1 | 0.99 (0.72-1.36) |  |  |
| Q4 vs Q1 | 1.16 (0.86-1.57) |  |  |
| Q5 vs Q1 | **1.51 (1.13-2.03)** |  | **1.05 (1.01-1.09)** |
| Age (years) | **1.08 (1.07-1.10)** | Age (years) | **1.08 (1.07-1.10)** |
| Male sex | **1.66 (1.28-2.16)** | Male sex | **1.68 (1.29-2.18)** |
| BMI (Kg/m^2^) | 0.99 (0.96-1.01) | BMI (Kg/m^2^) | 0.99 (0.96-1.01 ) |
| Physical activity Q2 vs Q1 | 0.98 (0.73-1.31) | Physical activity (MET/day) | **0.52 (0.31-0.90)** |
| Q3 vs Q1 | 0.79 (0.57-1.08) |  |  |
| Q4 vs Q1 | 1.00 (0.76-1.31) |  |  |
| Q5 vs Q1 | **0.60 (0.42-0.86)** |  |  |
| Mediterranean diet Q2 vs Q1 | 0.86 (0.63-1.16) | Mediterranean diet scale | 0.95 (0.90-1.02) |
| Q3 vs Q1 | 0.97 (0.73-1.29) |  |  |
| Q4 vs Q1 | 0.85 (0.62-1.16) |  |  |
| Q5 vs Q1 | 0.73 (0.52 -1.04) |  |  |
| Alcohol (gr/day) | 1.00 (1.00 -1.01) | Alcohol (gr/day) | 1.00 (1.00-1.01) |
| Social class Q2 vs Q1 | 1.04 (0.82-1.33) | Social class index | **0.95 (0.92-0.98)** |
| Q3 vs Q1 | 0.85 (0.61-1.17) |  |  |
| Q4 vs Q1 | 0.78 (0.56-1.10) |  |  |
| Q5 vs Q1 | **0.61 (0.41-0.92)** |  |  |
| Smoking (years) | **1.02 (1.01-1.02)** | Smoking (years) | **1.02 (1.01-1.02)** |
| LDL cholesterol (mg/dL) | 1.00 (0.99-1.00) | LDL colesterol (mg/dL) | 1.00 (0.99-1.00) |
| HDL cholesterol (mg/dL) | 1.00 (0.99-1.01) | HDL colesterol (mg/dL) | 1.00 (0.99-1.01) |
| Systolic blood pressure (mmHg) | 1.00 (1.00-1.01) | Systolic blood pressure (mmHg) | 1.00 (1.00-1.01) |
| Glycaemia (mg/dL) | **1.01 (1.00-1.01)** | Glycaemia (mg/dL) | **1.01 (1.00-1.01)** |
| Acute coronary syndrome | **1.52 (1.08-2.14)** | Acute coronary syndrome | **1.50 (1.07-2.11)** |
| Statins | 1.06 (0.82-1.36) | Statins | 1.07 (0.83-1.37) |
| Antihypertensives | 1.14 (0.90-1.46) | Antihypertensives | 1.14 (0.89-1.45) |
| Antidiabetics | 0.71 (0.50-1.01) | Antidiabetics | 0.71 (0.50-1.01) |

Table S7. The table presents a proportional hazard model for all-cause mortality, with social class being substituted by the participant's educational level.

|  | All-cause  deaths  HR (95% CI) |
| --- | --- |
| Resistin (ng/mL) Q2 vs Q1 | 1.11 (0.83-1.49) |
| Q3 vs Q1 | 0.94 (0.69-1.29) |
| Q4 vs Q1 | 1.15 (0.86-1.55) |
| Q5 vs Q1 | **1.53 (1.15-2.04)** |
| Age (years) | 1.09 (1.08-1.10) |
| Male sex | 2.52 (1.99-3.18) |
| BMI (Kg/m^2^) 25- 29 vs <25 | 0.59 (0.46-0.77) |
| ≥30 vs <25 | 0.67 (0.51-0.88) |
| Physical activity Q2 vs Q1 | 0.95 (0.72-1.26) |
| Q3 vs Q1 | 0.88 (0.65-1.20) |
| Q4 vs Q1 | 0.98 (0.75-1.28) |
| Q5 vs Q1 | 0.66 (0.47-0.94) |
| Low HDL cholesterol | 1.15 (0.93-1.41) |
| Mediterranean diet Q2 vs Q1 | 0.88 (0.66-1.18) |
| Q3 vs Q1 | 1.04 (0.79-1.37) |
| Q4 vs Q1 | 0.87 (0.64-1.19) |
| Q5 vs Q1 | 0.79 (0.56-1.12) |
| Moderate alcohol vs <1.5 g/d | 0.77 (0.61-0.98) |
| Excessive alcohol vs <1.5 g/d | 0.81 (0.58-1.12) |
| Educational level (L) L2 vs L1 | 0.79 (0.51-1.22) |
| L3 vs L1 | 0.62 (0.37-1.05) |
| L4 vs L1 | 0.40 (0.22-0.74) |
| Smoking | 1.69 (1.35-2.13) |
| Dyslipidemia | 1.00 (0.82-1.23) |
| Hypertension | 1.21 (0.97-1.50) |
| Diabetes | 2.06 (1.66-2.56) |
| Acute coronary syndrome | 1.71 (1.23-2.38) |

Table S8. The table presents a proportional hazard model for all-cause mortality, with social class and resistin being substituted by their multiple imputations values.

|  | All-cause  deaths  HR (95% CI) |
| --- | --- |
| Resistin (ng/mL) Q2 vs Q1 | 1.10 (0.97-1.23) |
| Q3 vs Q1 | 0.97 (0.85-1.10) |
| Q4 vs Q1 | 1.17 (1.04-1.32) |
| Q5 vs Q1 | **1.53 (1.36-1.72)** |
| Age (years) | 1.09 (1.09-1.10) |
| Male sex | 2.44 (2.21-2.68) |
| BMI (Kg/m^2^) 25- 29 vs <25 | 0.60 (0.54-0.67) |
| ≥30 vs <25 | 0.69 (0.61-0.77) |
| Physical activity Q2 vs Q1 | 0.94 (0.84-1.06) |
| Q3 vs Q1 | 0.88 (0.77-0.99) |
| Q4 vs Q1 | 1.00 (0.90-1.12) |
| Q5 vs Q1 | 0.66 (0.57-0.76) |
| Low HDL cholesterol | 1.13 (1.04-1.23) |
| Mediterranean diet Q2 vs Q1 | 0.87 (0.78-0.98) |
| Q3 vs Q1 | 1.01 (0.90-1.13) |
| Q4 vs Q1 | 0.85 (0.75-0.97) |
| Q5 vs Q1 | 0.78 (0.68-0.89) |
| Moderate alcohol vs <1.5 g/d | 0.76 (0.69-0.84) |
| Excessive alcohol vs <1.5 g/d | 0.84 (0.73-0.96) |
| Social class Q2 vs Q1 | 0.97 (0.88-1.06) |
| Q3 vs Q1 | 0.89 (0.78-1.01) |
| Q4 vs Q1 | 0.77 (0.67-0.88) |
| Q5 vs Q1 | 0.56 (0.48-0.66) |
| Smoking | 1.67 (1.52-1.83) |
| Dyslipidemia | 1.00 (0.92-1.08) |
| Hypertension | 1.24 (1.13-1.36) |
| Diabetes | 2.03 (1.85-2.21) |
| Acute coronary syndrome | 1.67 (1.46-1.92) |
